# Quantitative approach to analyze hyoid bone movement during swallowing by ultrasound: an integrative review

**DOI:** 10.1590/2317-1782/20232022002en

**Published:** 2023-07-17

**Authors:** Desiré Dominique Diniz de Magalhães, Jayne de Freitas Bandeira, Leandro Pernambuco

**Affiliations:** 1 Programa Associado de Pós-graduação em Fonoaudiologia, Universidade Federal da Paraíba - UFPB - João Pessoa (PB), Brasil.; 2 Departamento de Fonoaudiologia, Universidade Federal da Paraíba - UFPB - João Pessoa (PB), Brasil.

**Keywords:** Deglutition, Deglutition Disorders, Ultrasonography, Hyoid Bone, Movement

## Abstract

**Purpose:**

To synthesize the scientific knowledge on which measurements of hyoid bone movement during swallowing are obtained by ultrasonography and how to extract these measures.

**Research strategies:**

The PECO question and combinations of descriptors and keywords were formulated in the electronic databases Medline/PubMed, EMBASE, Web of Science, Scopus and Lilacs.

**Selection criteria:**

Articles that used ultrasonography to analyze measurements of hyoid bone movement during swallowing were included, regardless of language, year of publication, or presence of deglutition disorders.

**Data analysis:**

The included articles were analyzed for: year, study site, study design, population, sample size, equipment used, transducer positioning, measurements obtained, method of extraction, and reliability of measurements.

**Results:**

Twenty-six articles met the eligibility criteria. The most frequent measurement was hyoid movement maximum amplitude, followed by time and velocity. There was great variability in the study population, equipment used, positioning of the transducer and method of extraction of the measurements. Thus, it was not possible to find a standard model to extract the measures. The level of reliability was investigated in only eight articles.

**Conclusion:**

Amplitude, time and velocity are the measures of hyoid bone movement during swallowing that can be obtained by ultrasonography. There is no standardization on how to extract these measurements.

## INTRODUCTION

In oropharyngeal dysphagia there are changes in deglutition performance that can compromise the efficiency and safety of food intake^(1,2)^, with the possibility of affecting nutritional status, causing pulmonary complications, dehydration, malnutrition and, in more severe cases, pneumonia and death^(2)^. One of the precursor factors for the mechanism of this function to occur properly is the elliptical movement of the hyoid bone from the contraction of the suprahyoid muscles that promotes both lifting and hyolaryngeal anteriorization^(3,4)^.

The movement of the hyoid bone has an important role in the pharyngeal phase of deglutition, as it supports the protection of the lower airways by collaborating for glottis adduction and lowering of the epiglottis cartilage during lifting, as well as assists in the relaxation of the pharyngoesophageal segment during anteriorization^(4-8)^. Changes in the spatiotemporal synchrony of hyoid bone movement during deglutition may contribute to the incidence of pharyngeal residue (change in efficiency)^(5,6)^ and increased probability of inhalation (change in safety)^(7)^.

Therefore, in order to measure these clinical findings, studies present the importance of developing reliable quantitative methods to analyze the biomechanical events of deglutition^(7,8)^. These quantitative measures of hyoid bone movement during deglutition have already been widely used in diagnostic examinations such as Videofluoroscopic Swallowing Study (VFSS)^(9-13)^ and Ultrasonography (USG)^(6,8,14,15)^ Although VFSS is the instrumental method considered the reference standard because it allows the analysis of all deglutition phases, USG has been increasingly used as a complementary instrumental examination that allows the extraction of kinematic, temporal and interval measures related to the movement of the hyoid bone during deglutition^(16)^.

USG is a non-invasive procedure, without emission of ionizing radiation, which can be performed with patients in bed using real food without contrast^(17,18)^. This type of assessment can be used to estimate the efficiency of rehabilitation in individuals with dysphagia^(19)^ and allows a quantitative frame-by-frame analysis of the movement of the hyoid bone during deglutition^(20)^. The acoustic shadow generated by the hyoid bone in the USG examination makes it possible to obtain measures related to the course of the hyoid bone during deglutition^(21,22)^, such as, for example, temporal ^(8,14)^ and amplitude measures^(12,13,22,23)^. In order for this quantitative approach to occur in a valid, reliable and reproducible way in the scientific and clinical environment, it is recommended to standardize the measures and the extraction method from the examination^(24)^.

## OBJECTIVE

This study aims to summarize the situation of the scientific knowledge about which measures of hyoid bone movement during deglutition are obtained by ultrasonography and how to extract them.

## RESEARCH STRATEGIES

This is an integrative literature review, so it was not necessary to submit it to the evaluation of the Ethics and Human Research Committee of the institution. The methodological procedures for this type of review were followed according to the steps recommended in the literature.

The research question was formulated following the acronym PECO, which represents the elements Population, Exposure, Comparison and Outcome, respectively. The element (P) corresponds to “adults with or without deglutition disorders”; the second element (E) corresponds to “ultrasonography examination”; the third element (C) was not applied in this review; and the fourth element (O) was the “measures of hyoid bone movement during deglutition obtained by ultrasonography and its extraction method”. Thus, the research question was defined as: "What measures of hyoid bone movement during deglutition are obtained by ultrasonography and how are they extracted in adults with or without deglutition disorders?”

The database search took place in April 2021 and included the electronic databases Medline/PubMed, EMBASE, Web of Science, Scopus and Lilacs, all widely recognized in the health area. Furthermore, an additional search was carried out in the bibliographic references of the selected articles. The search strategies were made through the combinations between keywords and descriptors from *Medical Subjects Headings* (MeSH) and Health Sciences Descriptors (DeCS) (Appendix 1).

References were managed and duplicate articles were removed with the help of the EndNote Web® *software* (Clarivate, Thompson Reuters, New York, NY, USA). All searches were conducted on May 12, 2021. The application *Rayyan (Qatar Computing Research Institute*) was used as a tool for archiving, organizing and selecting articles among reviewers.

## SELECTION CRITERIA

For this review, the following inclusion criteria were applied: articles that used ultrasonography to analyze quantitative measures of hyoid bone movement during deglutition in adults aged 18 years or older, regardless of language, year of publication or presence of deglutition disorders. The following exclusion criteria were applied^(1)^: review articles, editorials, letters to the editor, conference annals, theses, and dissertations^(2)^; articles that evaluated hyoid bone movement through strictly descriptive analyses^(3)^; articles that included the adult and child population without separating the results by age group.

## DATA ANALYSIS

The articles were selected by two researchers independently, who compared their analyses and when disagreements occurred, these were resolved by a third evaluator. After extracting the articles from the databases and filtering out the duplicates, it was followed to the screening by reading the titles and abstracts, excluding those that did not meet the inclusion criteria.

The remaining articles that met the eligibility criteria or that generated some doubts were submitted to the reading of the full text. The reference lists of the selected articles were consulted in search of studies that had not been previously collected.

The articles that met the eligibility criteria were submitted to the extraction of the following data: author, year and place of the study, study design, population, sample size, objective, device used, positioning of the ultrasonography transducer, measures related to the movement of the hyoid bone during deglutition, method of extraction of the measures and reliability/accuracy of the measures.

## RESULTS

**Figure 1:**
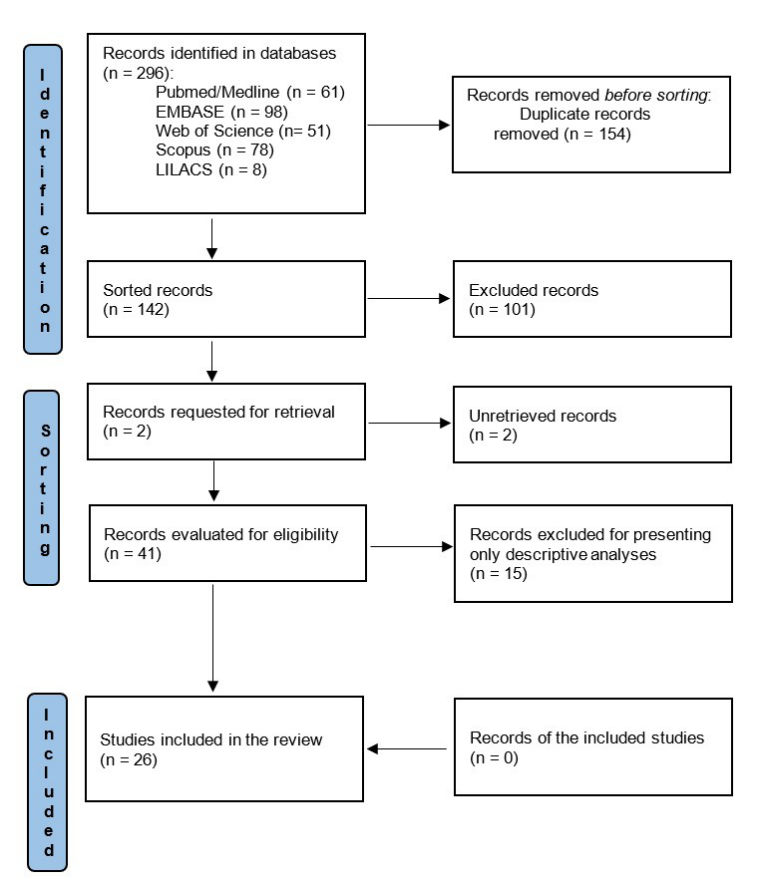
Flowchart of article selection

Using the aforementioned research strategies, 296 articles were located. After sorting by titles and abstracts, followed by reading the full text, the final sample consisted of 26 articles. This process is outlined in Figure 1.

The included studies were analyzed according to the data presented in the analysis matrix (Chart 1).

**Chart 1:** Analysis matrix of studies that used ultrasonography to evaluate measures of hyoid bone movement during deglutition

Author, year and place of study	Population and sample size	Device used	Positioning of ultrasonography transducer	Measures of hyoid bone movement during deglutition	Method for extraction of measures	Reliability of measures
Chen et al.^(26)^, 2020, Taiwan	98 seniors over 65 years old:	Unspecified	Submandibular area	Time = time interval from the beginning of the deglutition-related HB movement to the first moment of maximum amplitude in the course of the forward movement;	Unspecified	Not investigated
	47 with sarcopenia			Velocity = amplitude of the hyoid bone (HD) divided by the time interval.		
	47 without sarcopenia					
Chen et al.^(27)^, 2019, Taiwan	97 healthy adults without dysphagia	Self-designed model (LT701, LT701-000; LELTEK Corporation), connected to laptop, with curvilinear transducer (Convex Array, 3.75 MHz, P701-C04; LELTEK Corporation, Taipei City, Taiwan).	Submandibular area	Time interval from onset to amplitude	Using a two-axis coordinate system, the position of the HB relative to the mandible in each frame was represented as coordinate pairs. The distance between two coordinates before and during deglutition indicated the HB amplitude. The *software* employed was developed using the C Sharp programming language (Microsoft Visual Studio 2015; Microsoft, Redmond, WA, USA).	ICC = 0.845 (p <0.01)
				Amplitude		
				Velocity		
Chen et al.^(12)^, 2017, Taiwan	10 patients:	Self-designed model (LT701, LT701-000; LELTEK Corporation), connected to laptop, with curvilinear transducer (Convex Array, 3.75 MHz, P701-C04; LELTEK Corporation, Taipei City, Taiwan).	Submandibular area	Amplitude	The superior anterior margin of the fourth cervical vertebral body was used as a reference point for calculating HB movement. The initial position of the HB was marked and the movement relative to the reference point was calculated frame by frame to determine the amplitude.	Intra-examiner ICC of the two examiners = 0.996 and 0.959 (p <0.01), respectively.
	▪ 1 with stroke					Inter-examiner ICC = 0.892 (p <0.05)
	▪ 1 with neuromuscular disease					
	▪ 1 with traumatic brain injury					
	▪ 1 with chronic obstructive pulmonary disease					
	▪ 1 with spinal cord injury					
	▪ 1 with aspiration pneumonia					
	▪ 1 with gastroesophageal reflux disease					
Chi-Fishman e Sonies^(28)^, 2002, Estados Unidos	31 healthy adults:	Ultramark 9 model (Advanced Technology Laboratories; Bothell, WA) in combination with a Horita II time code generator (Mission Viejo, CA, model TRG-50PC) at 1/30-s speed, a Horita video distribution amplifier (VDA-50 Model) and a Sony U-matic VCR recorder (Tokyo, Japan, VO-5850 Model). 3.5-6.0 MHz wide openness annular array transducer.	Submandibular area	Duration of movement	Changes in position, such as points with cartesian X and Y coordinates, were measured. Each frame was a 640 × 480 matrix of pixels scaled to the centimeter unit at 39 pixels/centimeter based on the known depth. The reference point where X and Y were both equivalent to zero was in the upper left corner of each frame. All measurements were made with contrast enhancement using NIH Image version 1.61 with a custom macro.	Not investigated
	16 men			Amplitude/distance (where amplitude is the maximum movement achieved in the forward movement course only and distance is the total distance traveled in the forward and backward courses)		
	15 women			Velocity		
Chi-Fishman e Sonies^(29)^, 2002, Estados Unidos	30 healthy people stratified into three groups (young, middle-aged and elderly):	Ultramark 9 model (Advanced Technology Laboratories; Bothell, WA) in combination with a Horita II time code generator (Mission Viejo, CA, model TRG-50PC) at 1/30-s speed, a Horita video distribution amplifier (VDA-50 Model) and a Sony U-matic VCR recorder (Tokyo, Japan, VO-5850 Model). 3.5-6.0 MHz wide openness annular array transducer.	Submandibular area	Maximum amplitude	Changes in position, such as points with cartesian X and Y coordinates, were measured frame by frame by visual identification of the intersection of the anterior edge of the acoustic shadow of the HB and the upper edge of the muscles. Each frame was a 640 × 480 matrix of pixels scaled to the centimeter unit at 39 pixels/centimeter based on the known depth. All measurements were made with contrast enhancement using NIH Image version 1.61 with a custom macro.	
	15 men			Amplitude difference from start to finish		
	15 women			Total distance		
				Forward peak velocity		
				Peak backward velocity		
				Time from start to maximum		
				Time on maximum		
				Time from maximum to end		
				Total time		
				Time to peak velocity forward and backward		
				Stiffness index		
Costa et al.^(14)^, 2020, Brasil	40 women	LOGIQ P6 Model (GE Healthcare ®, Chicago, IL) in B mode, Vascular> Carotid adjustment, frame/second rate, 2.0-5.5 MHz convex linear array transducer.	Larynx (anterior area)	Lifting time	The videos were initially decomposed into frames using the converter *Free Video to JPG Converter* and subsequently analyzed using the *software* ImageJ to extract the measures of interest. Each measurement was the result of the time between the initial frame and the final frame of the respective event, in seconds. Each frame had 0.03 s (30 frames/second), so the number of frames for each measurement was calculated.	Inter-examiners ICC = 0.5 to 0.71
	20 submitted to total/partial thyroidectomy			Anteriorization time		Intra-examiner ICC (best examiner) = 0.75 to 0.92
	20 healthy women			Maximum amplitude time		
				Maximum amplitude holding time		
Dejaeger e Pelemans^(25)^, 1996, Bélgica	120 healthy individuals	Toshiba Sonolayer-L Sal 77A Model with a transducer of 3.75 MHz and a rate of 30 frames/second.	Unspecified	Time	The time was measured from the frame in which the HB moved anteriorly and superiorly from a rest position to the frame in which it returned to a stable rest position.	Not investigated
Feng et al.^(30)^, 2015, Estados Unidos	40 healthy young adults (20-40 years old):	Biosound MyLab25 Model (Esaote Group, Genoa, Italy), with an 18 MHz linear transducer.	Submandibular region	Maximum distance	Images of the movement from rest to maximum course were recorded and saved. The moving images have been revised and the frames have been frozen.	Not investigated
	20 men			Anterior amplitude		
	20 women			Superior amplitude		
Hsiao et al.^(20)^, 2012, Taiwan	60 patients with stroke	Self-designed model with a curvilinear transducer (BS3C673 Convex Array, 3.5 MHz, BSUS20-32C; Broadsound Corporation, Taiwan). Rate of 22.5 frames/second.	Submandibular area	Amplitude	The mandible was used as a reference point for calculating the movement of the HB. Using a two-axis coordinate system, the position of the HB relative to the mandible in each frame was represented as coordinate pairs. The distance between two coordinates before and during deglutition indicated the HB amplitude. The software used in this study was developed in MATLAB (v. 7.5.0, R2007b; The MathWorks, Inc., Boston, MA). The measurements were repeated 3 times for each participant to obtain the mean values for statistical analysis.	Intra-examiner ICC of the two examiners = 0.927 and 0.842, respectively;
	40 healthy individuals					Inter-examiner ICC = 0.806.
Kwak et al.^(5)^, 2018, Korea	20 patients with stroke and nasoenteral probe	Accuvix V10 model (Medison, Seoul, Korea) with a 3-6 MHz curved array transducer	Submandibular area	Time	Using the Kinovea v. 0.8.15 software, the time was automatically measured in milliseconds after the marker was placed on the HB. The area drawn by the marker is measured in pixel units using ImageJ 1.51 p. The analysis of the maximum movement is done by finding the greatest amplitude between any two points of the HB course during deglutition.	Not investigated
	25 patients with stroke and without nasoenteral probe			Amplitude		
	25 healthy adults without brain injury or dysphagia					
Lee et al.^(31)^, 2016, Korea	21 adults who did not inhale	LOGIQ E9 model (GE Healthcare, Milwaukee, WI, USA) with a 1-5 MHz curved probe.	Submandibular area	Amplitude	The examination was performed by measuring the distance between the acoustic shadows of the mandible and the HB at rest and during deglutition. The difference in distance was defined as amplitude of the HB. The HB amplitude's percentage was defined by the delta value.	Not investigated
	20 adults who had penetration					
	11 adults who inhale					
Li et al.^(43)^, 2016, Taiwan	20 individuals with post-stroke dysphagia:	Self-designed model with a curvilinear transducer (BS3C673 Convex Array, 3.5 MHz, BSUS20-32C; Broadsound Corporation, Taiwan). Rate of 22.5 frames/second.	Submandibular area	Amplitude	Unspecified	Not investigated
	10 received traditional therapy and *biofeedback* (experimental group)					
	10 received traditional deglutition therapy (control group)					
Macrae et al.^(23)^, 2012, Estados Unidos	5 healthy adults between the ages of 20 and 50 years old:	Scanner IU22 Model (Philips Ultrasound, Bothell, WA) with a 5-1 MHz curved array transducer.	Submandibular area	Amplitude	Amplitude was defined as the point at which the acoustic shadow intersects with the geniohyoid muscle. Each evaluator identified a "rest" frame before any deglutition-related oral movement of interest and a "maximum amplitude" frame. Electronic calipers were used to measure the distance between the reference point and the point of maximum amplitude. The quantification of the HB movement or the change from resting distance to amplitude distance were calculated as a percentage of the distance traveled from rest and as an absolute value of the distance traveled.	Inter-examiner ICC for percent change of hyoid position = 0.70
	02 men					Inter-examiner ICC for absolute change of hyoid position = 0.64
	03 women,					Intra-examiner ICC for percent change of hyoid position = 0.93
						Intra-examiner ICC for absolute change of hyoid position = 0.90
Matsuo e Matsuyama^(32)^, 2021, Japan	36 participants:	Color Doppler Ultrasound System JS2 Digital Model (SonoScape Medical Corp, Centennial, CO, USA). 5 to 12 MHz transducer	Larynx (thyroid cartilage on the left or right)	Amplitude in the lifting and return phases	The acquired images were analyzed using	Not investigated
	18 healthy elderly men				two-dimensional data analysis software (Dipp Motion Ver 1.1.31; DITECT Co., Tokyo, Japan). The measurement point was the lower anterior margin of the HB. The vertical and anteroposterior directions were considered as	
	18 male patients with stroke and diagnosis of neurogenic oropharyngeal dysphagia.				the x and y axes, respectively, and the distances moved in these directions were measured. The amplitude of movement of the HB and larynx were measured in the phases of lifting and lowering. The movement rate was calculated by dividing the bone movement (lifting phase) by the laryngeal movement (lifting phase) as the deglutition index.	
Matsuo et al.^(13)^, 2019, Japan	84 participants:	Color Doppler Ultrasound System JS2 Digital Model (SonoScape Medical Corp, Centennial, CO, USA). 5 to 12 MHz transducer	Larynx (thyroid cartilage on the left or right)	Amplitude	Amplitude was measured in both lifting and return phases defined as the period from the starting point to the position of maximum movement and the period from this position back to the rest position, respectively. The acquired images were converted into audio and video intermingle format and were analyzed using two-dimensional data analysis software (Dipp Motion Ver 1.1.31; DITECT Co, Tokyo, Japan). Markers were set at the lower anterior margin of the HB and the final upper part of the larynx, and the measurement points were automatically tracked in each frame using the tracking function of the analysis software. Vertical and anteroposterior directions were considered the x and y axes, respectively, and the distances moved in these directions were measured.	Not investigated
	42 young adults (20.3 ± 3.4 years old)					
	42 elderly (75.1 ± 10.6 years old).					
Perry et al.^(33)^, 2017, New Zealand	24 healthy participants (age 51-84 years old):	ACUSON Antares TM Model (ACUSON Antares TM 5.0 Premium Edition; Siemens Healthcare, Malvern, PA, USA) in 2D imaging function, mode B.	Submandibular area	Amplitude	For amplitude, two reference points were identified: (1) the intersection of the posterior edge of the shadow of the mental symphysis and the inferior portion of the geniohyoid muscle; and (2) the intersection of the anterior edge of the HB's bone shadow and the superior portion of the geniohyoid muscle. The frame that showed the greatest distance between these references (HB at rest) and the frame where the distance was smaller (maximum course). The distance between the two reference points was measured for rest and maximum amplitude. Images were imported into a Digital Imaging and Communications in Medicine (DICOM) viewer program (OsiriX MD; Pixmeo SARL, Bernex, Switzerland) on a computer (iMac; Apple Inc., Cupertino, CA, USA) for analysis.	Not investigated
	11 men					
	13 women					
Rocha et al.^(34)^, 2015, Brazil	100 healthy individuals stratified into 04 groups with 25 people each:	DP 6600 Model, micro-convex transducer coupled to	Submandibular area	Amplitude	The images were captured and subsequently analyzed using AAA (Articulate Assistant Advanced) Software. The amplitude between the lower part of the hyoid and the insertion of the mylohyoid muscle from the resting position was considered for maximum anteriorization.	Not investigated
	20 to 30 years old	a computer and head stabilizer.				
	31 to 40 years old					
	41 to 50 years old					
	51 to 60 years old					
Shawker et al.^(36)^, 1984, USA	10 healthy individuals (average age 24.8 years old)	Mechanical sector scanner model (Advanced Technology Laboratory) with 3mhz transducer, 80° sector angle and 30 frames/second rate.	Submandibular area	Time	Unspecified	Not investigated
	04 men					
	06 women					
Sonies et al.^(36)^, 1988, USA	47 healthy individuals (ages between 18-75 years old)	Mechanical sector scanner model (Advanced Technology Laboratory) with 3mhz transducer, 80° sector angle and 30 frames/second rate.	Submandibular area	Time	The duration measures of each deglutition were made from a frame-by-frame analysis of the videos, using a 10-second Sony video card. The time was measured from the moment the HB first moved anteriorly and superiorly from rest until its return to the stable rest position. The HB's movement was classified into four distinct phases and each phase was measured: from rest to anterior amplitude; time that the HB remained stable in the anterior amplitude; return to rest and total time.	

Sonies et al.^(37)^, 1996, USA	6 healthy volunteers:	Scanner ATL Ultramark 9 HDI Model	Submandibular area	Time	The images were scanned using a Scion LG-3 frame capture card on a Macintosh computer. The time intervals between markers A and B, B and C, and C and D were measured. A corresponds to the beginning of the HB movement, B indicates maximum lifting and beginning of the anterior movement, C corresponds to the anterior amplitude and beginning of the turning movement, and D indicates the completion of the return to the original rest position. From these measures, the durations for different phases of the movement were determined. The total movement time was calculated by the interval from A to D. The HB course was determined by tracking the movement in the HB muscle insertion region. The position of the HB during each deglutition was traced showing the way. A triangular course was used as a model to adjust the data, which allowed to calculate the movements during all phases.	Not investigated
	04 men	(Advanced Technology Laboratories, Inc., Bothell,		Amplitude		
	02 women	WA, USA). Curved array transducer with operating frequency of 5.0-9.0 MHz and a Doppler frequency of 5.0 MHz.		Velocity		
Steele et al.^(38)^, 2012, Canada	20 healthy young adults (age 20 to 39 years old:	GE LOGIQ alpha Model 100 MP Scanner (General Electric Medical	Submandibular area (no precise description of positioning)	Time	The recordings were analyzed in a similar way (frame by frame) to identify the temporal limits of movement of the acoustic shadow as follows: H1: First video frame showing anterior movement	Not investigated
	ten men	Systems, WI, USA) with 6.5 MHz E72 micro-convex transducer. Rate of 22.5 frames/second.			of the acoustic shadow during a deglutition; H2: final video frame before the posterior movement of the acoustic shadow during a deglutition; H3: First video frame showing the arrival of the HB shadow back in a stable resting position after swallowing. All values were converted to milliseconds to allow calculation of three time difference measurements.	
	ten women					
Stone and Shawker^(39)^, 1986, USA.	6 healthy adult women (ages between 20-40 years old).	Advanced Technology Laboratories, Inc. Model (Bellevue, WA). Mechanical sector scanner with rotating panel	Submandibular area	Time	The duration of the lifting, holding and return phases of each stage of laryngeal movement was determined by observing the HB movement and its acoustic shadow. The time was timed to hundredths of a second and digitally inserted into the videotape, marking each frame with a separate time and facilitating the analysis of the measures.	Not investigated
		head with three 3 MHz transducers spaced at 120 ~ around a				
		central axis				
Winiker et al.^(40)^, 2021,	20 healthy adults.	Clarius ™ Curvilinear Model (C3, Clarius, Burnaby, British Columbia, Canada; frequency range: 2-6 MHz,	Submandibular area	Amplitude	The amplitude peak position was defined as the frame with the shortest distance between the acoustic shadow of the HB and the acoustic shadow of the mental spine of the mandible. The extent of movement of the HB was expressed as a percentage of the distance in amplitude since rest (rest distance between the mental spine of the mandible and the HB - maximum distance between the two structures) / rest distance between the two structures) × 100.	Intra-examiner ICC = 0.25 (0.00-0.78)
		depth: 3-30 cm, size: 169 mm × 105 mm × 41 mm).				Inter-examiner ICC = 0.53 (0.01-0.83)
Yabunaka et al.^(41)^, 2009, Japan	15 healthy volunteers (average age 34.9 ± 9.3).	Xario Model (Toshiba Medical Systems, Tokyo, Japan) with	Larynx	Time	The images were analyzed by *ImageJ software* and the recordings were viewed at 30 frames/second for 3 seconds. Frames of the moving Image were analyzed and the amplitude of movement of the HB from the resting point was measured considering the X axis, horizontal migration length; and the Y axis, perpendicular migration length. The time intervals between markers A and B, B and C, and C and D were measured. A corresponds to the beginning of the HB movement, B indicates maximum lifting and beginning of the anterior movement, C corresponds to the anterior amplitude and beginning of the turning movement, and D indicates the completion of the return to the original rest position. The total movement time was calculated by the interval from A to D.	Not investigated
		curved array transducers (3.5-7.0 MHz) (PVT-674BT,		Amplitude		
		Toshiba).				
Yabunaka et al.^(42)^, 2010, Japan	30 healthy volunteers:	Xario Model (Toshiba Medical Systems, Tokyo, Japan) with	Larynx	Time	The images were analyzed by ImageJ software and the recordings were viewed at 30 frames/second for 3 seconds. Frames of the moving Image were analyzed and the amplitude of movement of the HB from the resting point was measured considering the X axis, horizontal migration length; and the Y axis, perpendicular migration length. The time intervals between markers A and B, B and C, and C and D were measured. A corresponds to the beginning of the HB movement, B indicates maximum lifting and beginning of the anterior movement, C corresponds to the anterior amplitude and beginning of the turning movement, and D indicates the completion of the return to the original rest position. The total movement time was calculated by the interval from A to D. The maximum HB lifting point was measured from A to C in all individuals.	Not investigated
	15 men	curved array transducers (3.5-7.0 MHz) (PVT-674BT,		Amplitude		
	15 women	Toshiba).				
Cordaro and Sons^(19)^, 1993, USA	A healthy male individual	Ultramark 9 Model (Advanced Technology Laboratories; Bothell, WA) with a curved array transducer (IYT).	Submandibular area (no precise description of positioning)	Amplitude	The images were spatially calibrated by the centimeter scale displayed by the equipment by counting the number of pixels between the scale markers. The HB coordinates extracted from the videofluorographic images were transformed into the coordinate system of the USG images frame by frame to make a direct quantitative comparison of the position and course of the HB. This was achieved by extracting the center of the curved array of the USG transducer directly from the videofluorographic image.	Not investigated

Among the publications included, two^(5,25)^ were classified as a case-control, one^(26)^ as cohort and 23^(12-14,19,20,23,27-43)^ as cross-sectional. Most of the articles were from North America ^(19,23,28-30,35-37,39)^ and Asia^(5,12,13,20,26,27,31,32,41-43)^.

There was a higher concentration of studies published between 2011 and 2021^(5,12,13,14,20,23,26,27,30-34,38,40,42,43)^, followed by the period between 2000 and 2010^(28,29,41)^, 1990 to 1999^(19,25,37)^ and 1980 to 1989^(35,36,39)^. The sample size varied from one^(19)^ and 120^(25)^ participants and most studies were conducted only with individuals without dysphagia or associated disease^(13,19,23,25-30,33-42)^. Half of the articles included did not specify the absolute frequency of the sample according to sex and, in those that included the information^(14,19,23,28-30,32,33,35,37-39,42)^, the groups were mostly mixed.

There was high variability regarding the equipment and the types and frequencies of the transducers used to perform the ultrasonography examination, with similarity only when the articles were from the same research group, which made it impossible to establish any standardization regarding these parameters. The positioning of the transducer was more frequent in the submandibular area^(5,12,19,20,23,26-31,33-40,43^, followed by the laryngeal area^(13,14,32,41,42)^. One study did not specify the positioning^(25)^.

The most investigated measures, in ascending order of frequency, were amplitude^(5,12,13,19,20,23,27-34,37,40-43)^, time ^(5,14,25-29,35-39,41,42)^, and velocity ^(26,-29,37)^. However, the methods for extraction were heterogeneous, with the use of different *softwares* to support the analysis of images decomposed into frames, and different methods of marking anatomical points of reference (Chart 1). The level of reliability was investigated in only six articles^(12,14,20,23,27,40)^, mostly through the intraclass correlation coefficient (ICC) with results ranging from moderate to excellent (Chart 1).

## DISCUSSION

This review summarized which measures of hyoid bone movement during deglutition are obtained by USG in adults with or without dysphagia, as well as their extraction methods. It was possible to identify more studies measuring movement amplitude, followed by time and velocity. In addition, the lack of standardization of the methods for obtaining and analyzing these measures was evidenced.

The results showed that the use of USG to analyze measures of hyoid bone movement during deglutition has been increasingly recurrent over the decades, mainly in studies in the United States, although other countries also stand out. This finding indicates increased interest of researchers and clinicians in finding instruments that are still not widespread and that can add benefits to the management of patients with dysphagia. In addition, it is necessary to consider that this scenario is also influenced by the momentum of accelerated technological advances in the health area in recent years, which allows techniques, procedures, and instruments to be developed or improved to be incorporated into clinical and research practice.

Although these aspects are positive, this study showed that the use of USG to measure the movement of the hyoid bone during deglutition does not yet have standardized procedures. Although the use of USG with quantitative approach to evaluate deglutition-related aspects has been described since the 80s^(35,36,39)^, its transposition into clinical practice remains challenging, considering that it is not a procedure usually explored for this purpose in the training of professionals who work with dysphagia^(24)^.

As for the quantitative parameters investigated in the studies included in this review, the measures of time, velocity and amplitude of movement, especially the latter, stand out as the main outcomes of the ultrasonography examination. However, the equipment used and the methods for obtaining and analyzing the measures are still quite heterogeneous or described in an incipient way, which compromises reproducibility and makes it difficult to transpose the examination to the clinical context.

The heterogeneity of the equipment used, including transducers, is a characteristic that limits comparisons between studies, given that each device has a different visualization rate, matrix and frequency, which can impact the marking of structures of interest. Regarding the positioning of the transducer, it can be concluded that the laryngeal and submandibular areas were the most used, the latter being the most frequent. Although in the same area, the anatomical markers may be different and interfere with the result of the image that will be analyzed. It is noteworthy that among the few studies that estimated the reliability^(12,14,20,23,27,40)^, the highest levels were among those that positioned the transducer in the submandibular area. However, it is not possible to prove whether there is a direct relationship between transducer positioning and reliability only from the data in this review.

The measure of maximum amplitude achieved by the hyoid bone during deglutition was the most investigated, although the reference points for obtaining this measure have been different. In addition to markers in the structure of the hyoid bone itself or in the acoustic shadow generated by it in the ultrasonography image, the mandible ^(20,27,31,40)^, the cervical vertebral body^(44)^ and adjacent musculature were used^(23,29,33,34)^. Something that may explain the maximum amplitude being the most investigated measure is that it directly reflects how far the hyoid bone is able to move away from its resting point while the individual swallows. This data is traditionally considered relevant in the diagnosis of oropharyngeal dysphagia, as it is related to the integrity of the lower airways protection and *clearance* pharyngeal^(3-8)^. In addition, among the measures found, amplitude is the simplest to be analyzed in operational terms, which may have favored its more frequent use.

With regard to temporal measures, it can be observed that the most investigated measure by the studies was the time interval between the beginning of the movement of the hyoid bone for deglutition until its maximum amplitude in the course^(5,14,25-29,35-39,41,42)^. It is worth highlighting this measure, because it is one of the indicators for the risk of penetration or inhalation^(6)^, since the decrease in amplitude time can compromise the movement of the epiglottis impairing the protection of the airway and, therefore, also hindering the opening of the pharyngoesophageal segment for passage of the food bolus^(44)^.

Most of the studies included were conducted with healthy individuals and investigated the measures regarding age, volume and consistency of the food bolus, since deglutition undergoes changes with advancing age^(44)^ and it is relevant to analyze the interrelationships between kinematic parameters to understand how motor control strategies occur by adjusting the volume and consistency^(29)^.

The levels of reliability found, although acceptable, are not comparable between studies, since the methods are different. It is noteworthy that the analysis of ultrasonography images is perceptual-visual, requires training and most studies performed the markings and analyses with non-automated processes. All these aspects constitute biases for the inter- and intra-evaluator reliability levels. In this review, we did not evaluate the outcomes of the measures, but we emphasize that they should be analyzed and interpreted considering the characteristics of the population studied.

## CONCLUSION

The most investigated measure of hyoid bone movement during deglutition using ultrasonography is amplitude, followed by time and velocity. The method for extracting the measures is not standardized with regard to the equipment used, transducer positioning and image analysis procedures.

## Appendix 1 Search strategies used in databases

**Table:**

Databases	Descriptors	Results
Pubmed/Medline	((“deglutition”[MeSH Terms] OR “deglutition”[All Fields] OR “deglutitions”[All Fields] OR “swallowing”[All Fields] OR “swallowings”[All Fields] OR “swallow”[All Fields] OR “swallows”[All Fields] OR “deglutition disorders”[MeSH Terms] OR “deglutition disorders”[All Fields] OR “deglutition disorder”[All Fields] OR “swallowing disorder”[All Fields] OR “swallowing disorders”[All Fields] OR “dysphagia”[All Fields]) AND (“hyoid bone”[MeSH Terms] OR “hyoid”[All Fields]) AND (“ultrasonography”[MeSH Terms] OR “ultrasonography”[All Fields] OR “diagnostic ultrasound”[All Fields] OR “ultrasound”[All Fields] OR “ultrasounds”[All Fields] OR “ultrasound imaging”[All Fields] OR “ultrasonic imaging”[All Fields] OR “medical sonography”[All Fields] OR “ultrasonographic imaging”[All Fields] OR “echography”[All Fields] OR “ultrasonic diagnoses”[All Fields] OR “ultrasonic diagnosis”[All Fields] OR “ultrasonics”[All Fields] OR “sonographic”[All Fields]))	61 results
EMBASE	(‘dysphagia’/exp OR ‘swallowing’/exp) AND (‘echography’/exp OR ‘ultrasound’/exp) AND (‘hyoid bone’/exp)	98 results
Web of Science	(TS=((“deglutition” OR “deglutitions” OR “swallowing” OR “swallowings” OR “swallow” OR “swallows” OR “deglutition disorders” OR “deglutition disorder” OR “swallowing disorder” OR “swallowing disorders” OR “dysphagia”) AND (“hyoid bone” OR “hyoid”) AND (“ultrasonography” OR “diagnostic ultrasound” OR “ultrasound” OR “ultrasound imaging” OR “ultrasonic imaging” OR “medical sonography” OR “ultrasonographic imaging” OR “echography” OR “ultrasonic diagnoses” OR “ultrasonic diagnosis” OR “ultrasonics” OR “sonographic”)))	51 results
Scopus	(TITLE-ABS-KEY (deglutition OR deglutitions OR swallowing OR swallowingsOR swallow OR swallows OR “deglutition AND disorders” OR “deglutition AND disorder” OR “swallowing AND disorder” OR “swallowing AND disorders” OR dysphagia) AND TITLE-ABS-KEY (“hyoid AND bone” OR “hyoid” ) AND TITLE-ABS-KEY (ultrasonography OR “diagnostic AND ultrasound” OR ultrasound OR ultrasounds))	78 results
LILACS	(deglutition OR “deglutition disorders” OR deglutição OR deglución OR “transtornos de deglutição” OR “trastornos de deglución” OR “troubles de la deglutition” OR deglutition) AND (“hyoid bone” OR “osso hioide” OR “hueso hioides” OR “os hyoide”) AND (Ultrasonography OR ultrasonografía OR échographie OR ultrassonografia)	8 results

**Caption:** ICC: intraclass correlation coefficient; HB: hyoid bone; USG: ultrasonograph
