# Supplementary material for: Quantitative approach to analyze hyoid bone movement during swallowing by ultrasound: an integrative review
Source: Codas. 2023 Jul 17;35(4):e20220002. doi: 10.1590/2317-1782/20232022002en (PMC10446754; doi:10.1590/2317-1782/20232022002en)
Supplement: Translation

# Translation: Abordagem quantitativa por ultrassonografia para análise do movimento do osso hioide durante a deglutição: revisão integrativa

**DOI:** 10.1590/2317-1782/20232022002pt

**Authors:** Desiré Dominique Diniz de Magalhães, Jayne de Freitas Bandeira, Leandro Pernambuco

**Affiliations:** 1 Programa Associado de Pós-graduação em Fonoaudiologia, Universidade Federal da Paraíba - UFPB - João Pessoa (PB), Brasil.; 2 Departamento de Fonoaudiologia, Universidade Federal da Paraíba - UFPB - João Pessoa (PB), Brasil.

**Keywords:** Deglutição, Transtornos de Deglutição, Ultrassonografia, Osso Hioide, Movimento

## Abstract

**Objetivo:**

Sintetizar o estado do conhecimento científico sobre quais medidas do movimento do osso hioide durante a deglutição são obtidas pela ultrassonografia e como extraí-las.

**Estratégia de pesquisa:**

A pergunta PECO e as combinações de descritores e palavras-chave foram formuladas nas bases de dados eletrônicas Medline/PubMed, EMBASE, Web of Science, Scopus and Lilacs.

**Critérios de seleção:**

Foram incluídos os artigos que utilizaram a ultrassonografia para analisar as medidas de movimento do osso hioide durante a deglutição, independentemente do idioma, ano de publicação ou presença de alteração na deglutição.

**Análise dos dados:**

Os artigos incluídos foram analisados quanto: ano, local do estudo, desenho do estudo, população, tamanho da amostra, equipamento utilizado, posicionamento do transdutor, medidas obtidas, método de extração e confiabilidade das medidas.

**Resultados:**

Vinte e seis artigos cumpriram os critérios de elegibilidade. A medida mais frequente foi a de amplitude máxima do movimento, seguida de tempo e velocidade. Houve grande variabilidade quanto à população de estudo, equipamentos utilizados, posicionamento do transdutor e método de extração das medidas, não sendo possível estabelecer padronização. O nível de confiabilidade foi investigado em apenas oito artigos.

**Conclusão:**

Amplitude, tempo e velocidade são as medidas do movimento do osso hioide durante a deglutição que podem ser obtidas por ultrassonografia. Não há padronização dos métodos de extração dessas medidas.

## INTRODUÇÃO

Na disfagia orofaríngea ocorrem mudanças no desempenho da deglutição que podem comprometer a eficiência e segurança na ingestão alimentar^(1,2)^, com possibilidade de acometer o estado nutricional, acarretar complicações pulmonares, desidratação, desnutrição e, em casos mais graves, pneumonia e óbito^(2)^. Um dos fatores precursores para que o mecanismo dessa função aconteça de modo adequado é o movimento elíptico do osso hioide a partir da contração da musculatura suprahioidea que promove tanto a elevação como a anteriorização hiolaríngea^(3,4)^.

O movimento do osso hioide possui um importante papel na fase faríngea da deglutição, pois dá suporte à proteção de vias aéreas inferiores ao colaborar para a adução glótica e abaixamento da cartilagem epiglote durante a elevação, assim como auxilia no relaxamento do segmento faringoesofágico durante a anteriorização^(4-8)^. Alterações na sincronia espaço-temporal do movimento do osso hioide durante a deglutição podem contribuir para a incidência de resíduos faríngeos (alteração na eficiência)^(5,6)^ e aumento da probabilidade de aspiração (alteração na segurança)^(7)^.

Portanto, com a finalidade de dimensionar esses achados clínicos, estudos apresentam a importância de desenvolver métodos quantitativos confiáveis para analisar os eventos biomecânicos da deglutição^(7,8)^. Essas medidas quantitativas de movimento do osso hioide durante a deglutição já tem sido amplamente utilizadas em exames de diagnóstico como a Videofluoroscopia da Deglutição (VFD)^(9-13)^ e a Ultrassonografia (USG)^(6,8,14,15)^ Embora a VFD seja o método instrumental considerado padrão de referência por permitir a análise de todas as fases da deglutição, a USG tem sido cada vez mais utilizada como um exame instrumental complementar que permite extrair medidas cinemáticas, temporais e de intervalo relacionadas ao movimento do osso hioide durante a deglutição^(16)^.

A USG é um procedimento não invasivo, sem emissão de radiação ionizante, que pode ser realizado com pacientes no leito utilizando alimentos reais sem contraste^(17,18)^. Este tipo de avaliação pode ser utilizado para estimar a eficiência da reabilitação em indivíduos com disfagia^(19)^ e permite realizar uma análise quantitativa quadro a quadro do movimento do osso hioide durante a deglutição^(20)^. A sombra acústica gerada pelo osso hioide no exame de USG possibilita a obtenção de medidas relacionadas à excursão do osso hioide durante a deglutição^(21,22)^, como, por exemplo, medidas temporais^(8,14)^ e de amplitude^(12,13,22,23)^. Para que essa abordagem quantitativa ocorra de modo válido, confiável e reprodutível no meio científico e clínico é recomendável padronizar as medidas e o método de extração a partir do exame^(24)^.

## OBJETIVO

O objetivo deste estudo é sintetizar o estado do conhecimento científico sobre quais medidas do movimento do osso hioide durante a deglutição são obtidas pela ultrassonografia e como extraí-las.

## ESTRATÉGIA DE PESQUISA

Trata-se de uma revisão da literatura do tipo integrativa, dessa forma, não foi necessário submetê-lo à avaliação do Comitê de Ética e Pesquisa em Seres Humanos da instituição. Foram seguidos os procedimentos metodológicos para este tipo de revisão de acordo com as etapas recomendadas na literatura.

A pergunta de pesquisa foi formulada seguindo o acrônimo PECO, que representa os elementos População, Exposição, Comparação e Desfecho, respectivamente. O elemento (P) corresponde aos “indivíduos adultos com ou sem alterações na deglutição”; o segundo elemento (E) corresponde ao “exame de ultrassonografia”; o terceiro elemento (C) não foi aplicado nesta revisão; e o quarto elemento (O) foram as “medidas do movimento do osso hioide durante a deglutição obtidas por ultrassonografia e o seu método de extração”. Assim, a pergunta de pesquisa foi definida como: “Quais as medidas do movimento do osso hioide durante a deglutição são obtidas por ultrassonografia e como elas são extraídas em indivíduos adultos com ou sem alterações na deglutição?”

A busca nas bases de dados ocorreu em abril de 2021 e incluiu as bases eletrônicas Medline/PubMed, EMBASE, Web of Science, Scopus e Lilacs, todas amplamente reconhecidas na área da saúde. Além disso, foi realizada uma busca adicional nas referências bibliográficas dos artigos selecionados. As estratégias de busca foram feitas por meio das combinações entre palavras-chave e descritores do *Medical Subjects Headings* (MeSH) e nos Descritores em Ciência da Saúde (DeCS) (Apêndice 1).

As referências foram gerenciadas e os artigos duplicados foram removidos com auxílio do *software* EndNote Web® (Clarivate, Thompson Reuters, New York, NY, USA). Todas as buscas foram realizadas em 12 de maio de 2021. O aplicativo*Rayyan (Qatar Computing Research Institute*) foi utilizado como uma ferramenta de arquivamento, organização e seleção dos artigos entre os revisores.

## CRITÉRIOS DE SELEÇÃO

Para esta revisão foram aplicados os seguintes critérios de inclusão: artigos que utilizaram a ultrassonografia para analisar as medidas quantitativas de movimento do osso hioide durante a deglutição em adultos com idade igual ou acima de 18 anos, independentemente do idioma, ano de publicação ou presença de alteração na deglutição. Foram aplicados os seguintes critérios de exclusão: (1) artigos de revisão, editoriais, cartas ao editor, anais de congresso, teses e dissertações; (2) artigos que avaliaram o movimento do osso hioide por meio de análises estritamente descritivas; (3) artigos que incluíram a população adulta e infantil sem separar os resultados por faixa etária.

## ANÁLISE DOS DADOS

Os artigos foram selecionados por dois pesquisadores independentemente, que compararam suas análises e quando ocorreram divergências, estas foram resolvidas por um terceiro avaliador. Após a extração dos artigos nas bases de dados e filtragem dos duplicados, seguiu-se para a triagem com leitura dos títulos e resumos, sendo excluídos aqueles que não atenderam aos critérios de inclusão.

Os demais artigos que atendiam aos critérios de elegibilidade ou que geraram alguma dúvida, foram submetidos à leitura do texto completo. As listas de referências dos artigos selecionados foram consultadas em busca de estudos que não haviam sido coletados anteriormente.

Os artigos que atendiam aos critérios de elegibilidade foram submetidos à extração dos seguintes dados: autor, ano e local do estudo, desenho do estudo, população, tamanho da amostra, objetivo, dispositivo utilizado, posicionamento do transdutor da ultrassonografia, medidas relacionadas ao movimento do osso hioide durante a deglutição, método de extração das medidas e confiabilidade/acurácia das medidas.

## RESULTADOS

Utilizando-se as estratégias de pesquisa supracitadas, foram localizados 296 artigos. Após a triagem por títulos e resumos, seguida pela leitura do texto completo, a amostra final foi constituída por 26 artigos^(5,12-14,19,20,23,25-43)^. Este processo está esquematizado na Figura 1.

**Figura 1:** Fluxograma de seleção dos artigos

Os estudos incluídos foram analisados de acordo com os dados apresentados na matriz de análise (Quadro 1).

**Quadro 1:** Matriz de análise dos estudos que utilizaram a ultrassonografia para avaliar medidas do movimento do osso hioide durante a deglutição

**Autor, ano e local do estudo**	**População e tamanho da amostra**	**Dispositivo utilizado**	**Posicionamento do transdutor da ultrassonografia**	**Medidas de movimento do osso hioide durante a deglutição**	**Método de extração das medidas**	**Confiabilidade das medidas**
Chen et al.^(26)^, 2020, Taiwan	98 idosos acima de 65 anos:	Não especificado	Região submandibular	Tempo = intervalo de tempo desde o início do movimento do OH relacionado à deglutição até o primeiro momento de amplitude máxima na trajetória do movimento para frente;	Não especificado	Não foi investigado
	47 sarcopênicos			Velocidade = amplitude do osso hióide (HD) dividido pelo intervalo de tempo.		
	47 não sarcopênicos					
Chen et al.^(27)^, 2019, Taiwan	97 adultos saudáveis sem disfagia	Modelo auto-projetado (LT701, LT701-000; LELTEK Corporation), conectada a laptop, com transdutor curvilíneo (Convex Array, 3,75 MHz, P701-C04; LELTEK Corporation, Taipei City, Taiwan).	Região submandibular	Intervalo de tempo do início à amplitude	Usando um sistema de coordenadas de dois eixos, a posição do OH em relação à mandíbula em cada quadro foi representada como pares de coordenadas. A distância entre duas coordenadas antes e durante a deglutição denotou a amplitude do OH. O *software* empregado foi desenvolvido utilizando a linguagem de programação C Sharp (Microsoft Visual Studio 2015; Microsoft, Redmond, WA, EUA).	CCI = 0,845 (p <0,01)
				Amplitude		
				Velocidade		
Chen et al.^(12)^, 2017, Taiwan	10 pacientes:	Modelo auto-projetado (LT701, LT701-000; LELTEK Corporation), conectada a laptop, com transdutor curvilíneo (Convex Array, 3,75 MHz, P701-C04; LELTEK Corporation, Taipei City, Taiwan).	Região submandibular	Amplitude	A margem anterior superior do quarto corpo vertebral cervical foi usada como ponto de referência para o cálculo do movimento do OH. A posição inicial do OH foi marcada e o movimento em relação ao ponto de referência foi calculado quadro a quadro para determinar a amplitude.	CCI intraexaminador dos dois examinadores = 0,996 e 0,959 (p <0,01), respectivamente.
	▪ 1 com AVC					CCI interexaminadores = 0,892 (p <0,05)
	▪ 1 com doença neuromuscular					
	▪ 1 com lesão cerebral traumática					
	▪ 1 com doença pulmonar obstrutiva crônica					
	▪ 1 com lesão medular					
	▪ 1 com pneumonia aspirativa					
	▪ 1 com doença de refluxo gastroesofágico					
Chi-Fishman e Sonies^(28)^, 2002, Estados Unidos	31 adultos saudáveis:	Modelo Ultramark 9 (Advanced Technology Laboratories; Bothell, WA) em combinação com um gerador de código de tempo Horita II (Mission Viejo, CA, Modelo TRG-50PC) a 1/30-s velocidade, um amplificador de distribuição de vídeo Horita (Modelo VDA-50) e um gravador de videocassete Sony U-matic (Tóquio, Japão, Modelo VO-5850). Transdutor de matriz anular de ampla abertura de 3,5-6,0 MHz.	Região submandibular	Duração do movimento	Mudanças de posição, como pontos com coordenadas cartesianas X e Y, foram medidas. Cada quadro era uma matriz de 640 × 480 de pixels dimensionada para a unidade de centímetros em 39 pixels/centímetro com base na profundidade conhecida. O ponto de referência onde X e Y eram ambos equivalentes a zero estava no canto superior esquerdo de cada quadro. Todas as medições foram feitas com realce de contraste por meio do NIH Image Versão 1.61 com uma macro customizada.	Não foi investigado
	16 homens			Amplitude/distância (onde a amplitude é o movimento máximo alcançado apenas na trajetória do movimento para frente e a distância é a distância total percorrida nas trajetórias para frente e para trás)		
	15 mulheres			Velocidade		
Chi-Fishman e Sonies^(29)^, 2002, Estados Unidos	30 pessoas saudáveis estratificadas em três grupos (jovens, meia idade e idosos):	Modelo Ultramark 9 (Advanced Technology Laboratories; Bothell, WA) em combinação com um gerador de código de tempo Horita II (Mission Viejo, CA, Modelo TRG-50PC) a 1/30-s velocidade, um amplificador de distribuição de vídeo Horita (Modelo VDA-50) e um gravador de videocassete Sony U-matic (Tóquio, Japão, Modelo VO-5850). Transdutor de matriz anular de ampla abertura de 3,5-6,0 MHz.	Região submandibular	Amplitude máxima	Mudanças de posição, como pontos com coordenadas cartesianas X e Y, foram medidas quadro a quadro por identificação visual da intersecção da borda anterior da sombra acústica do OH e a borda superior dos músculos. Cada quadro era uma matriz de 640 × 480 de pixels dimensionada para a unidade de centímetros em 39 pixels/centímetro com base na profundidade conhecida. Todas as medições foram feitas com realce de contraste por meio do NIH Image Versão 1.61 com uma macro customizada.	
	15 homens			Diferença de amplitude do início ao fim		
	15 mulheres			Distância total		
				Velocidade de pico à frente		
				Velocidade de pico para trás		
				Tempo do início ao máximo		
				Tempo no máximo		
				empo do máximo ao fim		
				Tempo total		
				Tempo para a velocidade de pico para frente e para trás		
				Índice de rigidez		
Costa et al.^(14)^, 2020, Brasil	40 mulheres	Modelo LOGIQ P6 (GE Healthcare ®, Chicago, IL) em modo B, ajuste Vascular> Carotídeo, taxa de frames/segundo, transdutor convexo de matriz linear de 2,0-5,5 MHz.	Laringe (região anterior)	Tempo de elevação	Os vídeos foram inicialmente decompostos em quadros usando o conversor *Free Video to JPG Converter* e posteriormente analisado usando o *software* ImageJ para extrair as medidas de interesse. Cada medição foi o resultado do tempo entre o quadro inicial e o quadro final do respectivo evento, em segundos. Cada quadro tinha 0,03 s (30 quadros/segundo), então, o número de quadros para cada medição foi calculado.	CCI interexaminadores = 0,5 a 0,71
	20 submetidas à tireoidectomia total/parcial			Tempo de anteriorização		CCI intraexaminador (melhor examinador) = 0,75 a 0,92
	20 mulheres saudáveis			Tempo máximo de amplitude		
				Tempo máximo de manutenção da amplitude		
Dejaeger e Pelemans^(25)^, 1996, Bélgica	120 indivíduos saudáveis	Modelo Toshiba Sonolayer-L Sal 77A com um transdutor de 3,75 MHz e uma taxa de 30 frames/segundo.	Não especificado	Tempo	O tempo foi medido a partir do quadro em que o OH se moveu anteriormente e superiormente de uma posição de repouso para o quadro no qual retornou a uma posição de repouso estável.	Não foi investigado
Feng et al.^(30)^, 2015, Estados Unidos	40 jovens adultos saudáveis (20-40 anos):	Modelo Biosound MyLab25 (Esaote Group, Gênova, Itália), com um transdutor linear de 18 MHz.	Região submandibular	Distância máxima	Imagens do movimento desde o repouso até a excursão máxima foram registradas e salvas. As imagens em movimento foram revisadas e os quadros congelados.	Não foi investigado
	20 homens			Amplitude anterior		
	20 mulheres			Amplitude superior		
Hsiao et al.^(20)^, 2012, Taiwan	60 pacientes com AVC	Modelo autoprojetado com um transdutor curvilíneo (BS3C673 Convex Array, 3,5 MHz, BSUS20-32C; Broadsound Corporation, Taiwan). Taxa de 22,5 frames/segundo.	Região submandibular	Amplitude	A mandíbula foi usada como ponto de referência para o cálculo do movimento do OH. Usando um sistema de coordenadas de dois eixos, a posição do OH em relação à mandíbula em cada quadro foi representada como pares de coordenadas. A distância entre duas coordenadas antes e durante a deglutição denota o movimento do OH. O software empregado neste estudo foi desenvolvido em MATLAB (v. 7.5.0, R2007b; The MathWorks, Inc., Boston, MA). As medidas foram repetidas 3 vezes para cada participante, para obtenção dos valores médios para análise estatística.	CCI intraexaminador dos dois examinadores = 0,927 e 0,842, respectivamente;
	40 indivíduos saudáveis					CCI interexaminador = 0,806.
Kwak et al.^(5)^, 2018, Coreia	20 pacientes com AVC e sonda nasoenteral	Modelo Accuvix V10 (Medison, Seul, Coréia) com um transdutor de matriz curva de 3-6 MHz	Região submandibular	Tempo	Usando o software Kinovea v.0.8.15, o tempo foi medido automaticamente em milissegundos após o marcador ter sido colocado no OH. A área desenhada pelo marcador é medida em unidades de pixel usando ImageJ 1.51p. A análise do movimento máximo é feita encontrando-se a maior amplitude entre quaisquer dois pontos da trajetória do OH durante a deglutição.	Não foi investigado
	25 pacientes com AVC sem sonda nasoenteral			Amplitude		
	25 adultos saudáveis sem lesões cerebrais ou disfagia					
Lee et al.^(31)^, 2016, Coreia	21 adultos que não aspiravam	Modelo LOGIQ E9 (GE Healthcare, Milwaukee, WI, EUA) com uma sonda curva de 1-5 MHz.	Região submandibular	Amplitude	O exame foi realizado medindo-se a distância entre as sombras acústicas da mandíbula e OH em repouso e durante a deglutição. A diferença na distância foi definida como amplitude do OH. A porcentagem de amplitude do OH foi definida pelo valor de delta.	Não foi investigado
	20 adultos que tiveram penetração					
	11 adultos que aspiravam					
Li et al.^(43)^, 2016, Taiwan	20 indivíduos com disfagia pós-AVC:	Modelo autoprojetado com um transdutor curvilíneo (BS3C673 Convex Array, 3,5 MHz, BSUS20-32C; Broadsound Corporation, Taiwan). Taxa de 22,5 frames/segundo.	Região submandibular	Amplitude	Não especificado	Não foi investigado
	10 receberam a terapia tradicional e *biofeedback* (grupo experimental)					
	10 receberam terapia de deglutição tradicional (grupo controle)					
Macrae et al.^(23)^, 2012, Estados Unidos	5 adultos saudáveis com idades entre 20 e 50 anos:	Modelo Scanner IU22 (Philips Ultrasound, Bothell, WA) com um transdutor de matriz curva de 5-1 MHz.	Região submandibular	Amplitude	A amplitude foi definida como o ponto em que a sombra acústica cruza com o músculo geniohioideo. Cada avaliador identificou um quadro de ''descanso'' antes de qualquer movimento oral relacionado à deglutição de interesse e um quadro de 'amplitude máxima'. Paquímetros eletrônicos foram usados ​​para medir a distância entre o ponto de referência e o ponto de máxima amplitude. A quantificação do movimento do OH ou a mudança da distância em repouso para a distância de amplitude foi calculada como uma porcentagem da distância percorrida do repouso e como valor absoluto da distância percorrida.	CCI interexaminador para a mudança percentual da posição hióide = 0,70
	02 homens					CCI interexaminador para a mudança absoluta da posição hióide = 0,64
	03 mulheres,					CCI intraexaminador para a mudança percentual da posição hióide = 0,93
						CCI intraexaminador para a mudança absoluta da posição hióide = 0,90
Matsuo e Matsuyama^(32)^, 2021, Japão	36 participantes:	Modelo Digital Color Doppler Ultrasound System JS2 (SonoScape Medical Corp, Centennial, CO, EUA). Transdutor de 5 a 12 MHz	Laringe (cartilagem tireoide à esquerda ou direita)	Amplitude nas fases de elevação e retorno	As imagens adquiridas foram analisadas usando	Não foi investigado
	18 idosos saudáveis do sexo masculino				software de análise de dados bidimensional (Dipp Motion Ver 1.1.31; DITECT Co., Tóquio, Japão). O ponto de medição foi a margem anterior inferior do OH. As direções vertical e ântero-posterior foram consideradas como	
	18 pacientes do sexo masculino com AVC e diagnóstico de disfagia orofaríngea neurogênica.				os eixos x e y, respectivamente, e as distâncias movidas nessas direções foram medidas. A amplitude do movimento do OH e laringe foi medido nas fases de elevação e descida. A taxa de movimento foi calculada dividindo o movimento ósseo (fase de elevação) pelo movimento laríngeo (fase de elevação) como índice da deglutição.	
Matsuo et al.^(13)^, 2019, Japão	84 participantes:	Modelo Digital Color Doppler Ultrasound System JS2 (SonoScape Medical Corp, Centennial, CO, EUA). Transdutor de 5 a 12 MHz	Laringe (cartilagem tireoide à esquerda ou direita)	Amplitude	A amplitude foi medida em ambas as fases de elevação e retorno definidas como o período do ponto de partida até a posição de movimento máximo e o período desta posição de volta à posição de repouso, respectivamente. As imagens adquiridas foram convertidas em formato de intercalação de áudio e vídeo e foram analisados ​​usando software de análise de dados bidimensional (Dipp Motion Ver 1.1.31; DITECT Co, Tóquio, Japão). Os marcadores foram definidos na margem anterior inferior do OH e a parte superior final da laringe, e os pontos de medição foram rastreados automaticamente em cada quadro usando a função de rastreamento do software de análise. Direções vertical e ântero-posterior foram considerados os eixos x e y, respectivamente, e o distâncias movidas nessas direções foram medidas.	Não foi investigado
	42 adultos jovens (20,3 ± 3,4 anos)					
	42 idosos (75,1 ± 10,6 anos).					
Perry et al.^(33)^, 2017, Nova Zelândia	24 participantes saudáveis (idade de 51-84 anos):	Modelo ACUSON Antares TM (ACUSON Antares TM 5.0 Premium Edition; Siemens Healthcare, Malvern, PA, EUA) na função de imagem 2D, modo B.	Região submandibular	Amplitude	Para a amplitude, dois pontos de referência foram identificados: (1) a intersecção da borda posterior da sombra da sínfise mental e a porção inferior do músculo geniohioideo; e (2) a intersecção da borda anterior da sombra óssea do OH e a porção superior do músculo geniohioideo. O quadro que mostrou a maior distância entre essas referências (OH em repouso) e o quadro onde a distância era menor (excursão máxima). A distância entre os dois pontos de referência foi medida para repouso e amplitude máxima. Imagens foram importadas em um programa visualizador de Digital Imaging and Communications in Medicine (DICOM) (OsiriX MD; Pixmeo SARL, Bernex, Suíça) em um computador (iMac; Apple Inc., Cupertino, CA, EUA) para análise.	Não foi investigado
	11 homens					
	13 mulheres					
Rocha et al.^(34)^, 2015, Brasil	100 indivíduos saudáveis ​​estratificados em 04 grupos com 25 pessoas cada:	Modelo DP 6600, transdutor micro-convexo acoplado a	Região submandibular	Amplitude	As imagens foram capturadas e, posteriormente, analisadas por meio do Software AAA (Articulate Assistant Advanced). Foi considerada a amplitude entre a parte inferior do hióide e a inserção do músculo milo-hioideo da posição de repouso para máxima anteriorização.	Não foi investigado
	20 a 30 anos	um computador e estabilizador de cabeça.				
	31 a 40 anos					
	41 a 50 anos					
	51 a 60 anos					
Shawker et al.^(35)^, 1984, Estados Unidos	10 indivíduos saudáveis (idade média de 24,8 anos)	Modelo scanner mecânico de setorização (Advanced Technology Laboratory) com transdutor de 3mhz, ângulo de setorização de 80° e taxa de 30 frames/segundo.	Região submandibular	Tempo	Não especificado	Não foi investigado
	04 homens					
	06 mulheres					
Sonies et al.^(36)^, 1988, Estados Unidos	47 indivíduos saudáveis ​​(idades entre 18-75 anos)	Modelo scanner mecânico de setorização (Advanced Technology Laboratory) com transdutor de 3mhz, ângulo de setorização de 80° e taxa de 30 frames/segundo.	Região submandibular	Tempo	As medidas de duração de cada deglutição foram feitas a partir de uma análise quadro a quadro dos vídeos, utilizando uma placa de vídeo Sony de 10 segundos. O tempo foi medido a partir do momento que o OH se moveu pela primeira vez anterior e superiormente do repouso até seu retorno à posição de repouso estável. O movimento do OH foi classificado em quatro fases distintas e cada fase foi medida: do repouso à amplitude anterior; tempo que o OH permaneceu estável na amplitude anterior; retorno ao repouso e tempo total.	

Sonies et al. ^(37)^, 1996, Estados Unidos	6 voluntários saudáveis:	Modelo scanner ATL Ultramark 9 HDI	Região submandibular	Tempo	As imagens foram digitalizadas usando uma placa de captura de quadros Scion LG-3 em um computador Macintosh. Foram medidos os intervalos de tempo entre os marcadores A e B, B e C e C e D. A corresponde o início do movimento do OH, B indica elevação máxima e início do movimento anterior, C corresponde à amplitude anterior e início do movimento de giro e D indica a conclusão do retorno para a posição de repouso original. A partir dessas medidas, foram determinadas as durações para diferentes fases do movimento. O tempo total do movimento foi calculado pelo intervalo de A a D. A trajetória do OH foi determinada pelo rastreamento do movimento na região de inserção muscular do OH. A posição do OH durante cada deglutição foi traçada mostrando o caminho. Uma trajetória triangular foi usado como modelo para ajustar os dados, o que permitiu calcular os movimentos durante todas as fases.	Não foi investigado
	04 homens	(Advanced Technology Laboratories, Inc., Bothell,		Amplitude		
	02 mulheres	WA, EUA). Transdutor de matriz curva com frequência operacional de 5,0-9,0 MHz e uma frequência Doppler de 5,0 MHz.		Velocidade		
Steele et al. ^(38)^, 2012, Canadá	20 adultos jovens saudáveis (idade de 20 a 39 anos:	Modelo GE LOGIQ alpha	Região submandibular (sem descrição precisa do posicionamento)	Tempo	As gravações foram analisadas de forma semelhante (quadro a quadro) para identificar os limites temporais de movimento da sombra acústica da seguinte forma: H1: Primeiro quadro de vídeo mostrando movimento anterior	Não foi investigado
	dez homens	Scanner de 100 MP (General Electric Medical			da sombra acústica durante uma deglutição; H2: quadro de vídeo final antes do movimento posterior da sombra acústica durante uma deglutição; H3: Primeiro quadro de vídeo mostrando a chegada da sombra do OH de volta em uma posição de descanso estável após engolir. Todos os valores foram convertidos em milissegundos para permitir o cálculo de três medidas de diferença de tempo.	
	dez mulheres	Systems, WI, EUA) com transdutor microconvexo E72 de 6,5 MHz. Taxa de 29,97 frames/segundos.				
Stone e Shawker ^(39)^, 1986, Estados Unidos.	6 mulheres adultas saudáveis (idades entre 20 e 40 anos).	Modelo Advanced Technology Laboratories, Inc. (Bellevue, WA). Scanner de setor mecânico com um painel rotativo cabeça com três transdutores de 3 MHz espaçados em 120 ~ em torno de um eixo central	Região submandibular	Tempo	A duração das fases de elevação, manutenção e retorno de cada etapa do movimento laríngeo foi determinada pela observação do movimento do OH e sua sombra acústica. O tempo foi cronometrado a centésimos de segundo e inserido digitalmente na fita de vídeo, marcando cada quadro com um tempo separado e facilitando a análise das medidas.	Não foi investigado
Winiker et al.^(40)^, 2021, Nova Zelândia	20 adultos saudáveis.	Modelo curvilíneo Clarius ™ (C3, Clarius, Burnaby, British Columbia, Canadá; faixa de frequência: 2-6 MHz,	Região submandibular	Amplitude	A posição de pico de amplitude foi definida como o quadro com menor distância entre a sombra acústica do OH e a sombra acústica da espinha mentual da mandíbula. A extensão do movimento do OH foi expressa como uma porcentagem da distância na amplitude desde o repouso (distância de repouso entre a coluna mentual da mandíbula e o OH - distância máxima entre os dois estruturas) / distância de repouso entre as duas estruturas) × 100.	CCI intraexaminador = 0.25 (0.00-0.78)
		profundidade: 3-30 cm, tamanho: 169 mm × 105 mm × 41 mm).				CCI interexaminador = 0.53 (0.01-0.83)
Yabunaka et al.^(41)^, 2009, Japão	15 voluntários saudáveis (idade média de 34,9 ± 9,3).	Modelo Xario (Toshiba Medical Systems, Tóquio, Japão) com	Laringe	Tempo	As imagens foram analisadas por *software ImageJ* e as gravações foram visualizadas a 30 quadros/segundo por 3 segundos. Quadros da imagem em movimento foram analisados e a amplitude de movimento do OH do ponto de repouso foi medido considerando o eixo X, comprimento de migração horizontal; e o eixo Y, comprimento de migração perpendicular. Mediram os intervalos de tempo entre A e B, B e C, C e D. A corresponde o início do movimento do OH, B indica elevação máxima e início do movimento anterior, C corresponde à amplitude anterior e início do movimento de giro e D indica a conclusão do retorno para a posição de repouso original. O tempo total do movimento foi calculado pelo intervalo de A a D.	Não foi investigado
		transdutores de matriz curva (3,5-7,0 MHz) (PVT-674BT,Toshiba).		Amplitude		
Yabunaka et al.^(42)^, 2011, Japão	30 voluntários saudáveis:	Modelo Xario (Toshiba Medical Systems, Tóquio, Japão) com	Laringe	Tempo	As imagens foram analisadas por software ImageJ e as gravações foram visualizadas a 30 quadros/segundo por 3 segundos. Quadros da imagem em movimento foram analisados e a amplitude de movimento do OH do ponto de repouso foi medido considerando o eixo X, comprimento de migração horizontal; e o eixo Y, comprimento de migração perpendicular. Mediram os intervalos de tempo entre A e B, B e C, C e D. A corresponde o início do movimento do OH, B indica elevação máxima e início do movimento anterior, C corresponde à amplitude anterior e início do movimento de giro e D indica a conclusão do retorno para a posição de repouso original. O tempo total do movimento foi calculado pelo intervalo de A a D. O ponto de elevação máxima do OH foi medido de A a C em todos os indivíduos.	Não foi investigado
	15 homens	transdutores de matriz curva (3,5-7,0 MHz) (PVT-674BT,		Amplitude		
	15 mulheres	Toshiba).				
Cordaro e Sonies^(19)^, 1993, Estados Unidos	Um indivíduo saudável do sexo masculino	Modelo Ultramark 9 (Advanced Technology Laboratories; Bothell, WA) com um transdutor de matriz curva (IYT).	Região submandibular (sem descrição precisa do posicionamento)	Amplitude	As imagens foram calibradas espacialmente pela escala centimétrica exibida pelo equipamento por meio da contagem do número de pixels entre os marcadores da escala. As coordenadas do OH extraídas das imagens videofluorográficas foram transformadas no sistema de coordenadas das imagens de USG quadro a quadro para fazer uma comparação quantitativa direta da posição e trajetória do OH. Isso foi conseguido extraindo o centro da matriz curva do transdutor de USG diretamente da imagem videofluorográfica.	Não foi investigado

Dentre as publicações incluídas, duas^(5,25)^ foram classificados como caso-controle, uma^(26)^ como coorte e 23^(12-14,19,20,23,27-43)^como transversal. A maior parte dos artigos foram de origem norte-americana^(19,23,28-30,35-37,39)^e asiática^(5,12,13,20,26,27,31,32,41-43)^.

Houve maior concentração de estudos publicados entre 2011 e 2021^(5,12,13,14,20,23,26,27,30-34,38,40,42,43)^, seguido pelo período entre 2000 e 2010^(28,29,41)^, 1990 a 1999^(19,25,37)^ e 1980 a 1989^(35,36,39)^. O tamanho da amostra variou entre um^(19)^ e 120^(25)^ participantes e a maioria dos estudos foi realizada apenas com indivíduos sem disfagia ou doença associada^(13,19,23,25-30,33-42)^. Metade dos artigos incluídos não especificou a frequência absoluta da amostra de acordo com o sexo e naqueles que incluíram a informação^(14,19,23,28-30,32,33,35,37-39,42)^ os grupos foram mistos em sua maioria.

Houve alta variabilidade quanto aos equipamentos e os tipos e frequências dos transdutores utilizados para realizar o exame de ultrassonografia, havendo semelhança apenas quando os artigos eram do mesmo grupo de pesquisa, o que inviabilizou estabelecer qualquer padronização em relação a esses parâmetros. Já o posicionamento do transdutor foi mais frequente na região submandibular ^(5,12,19,20,23,26-31,33-40,43)^, seguido da região laríngea^(13,14,32,41,42)^. Um estudo não especificou o posicionamento^(25)^.

As medidas mais investigadas, por ordem crescente de frequência, foram amplitude^(5,12,13,19,20,23,27-34,37,40-43)^, tempo^(5,14,25-29,35-39,41,42)^, e velocidade^(26,-29,37)^. Entretanto, os métodos para extração foram heterogêneos, com uso de diferentes *softwares* de apoio à análise de imagens decompostas em quadros, e distintos métodos de marcação de pontos de referência anatômicos (Quadro 1). O nível de confiabilidade foi investigado em apenas seis artigos ^(12,14,20,23,27,40)^, em sua maioria por meio do coeficiente de correlação intraclasse (CCI) com resultados que variaram de moderado a excelente (Quadro 1).

## DISCUSSÃO

Esta revisão sintetizou quais as medidas do movimento do osso hioide durante a deglutição são obtidas por USG em pessoas adultas com ou sem disfagia, assim como seus métodos de extração. Foi possível identificar maior frequência de estudos que medem amplitude do movimento, seguido por tempo e velocidade. Além disso, foi evidenciada a ausência de padronização dos métodos para obtenção e análise dessas medidas.

Os resultados apontaram que o uso da USG para analisar medidas de movimento do osso hioide durante a deglutição tem sido cada vez mais recorrente ao longo das décadas, principalmente em estudos nos Estados Unidos, ainda que outros países também se destaquem. Esse achado denota o aumento do interesse de pesquisadores e clínicos em encontrar instrumentos ainda pouco difundidos que possam agregar benefícios ao manejo do paciente disfágico. Além disso, é necessário ponderar que esse cenário também é influenciado pelo impulso do acelerado avanço tecnológico na área da saúde nos últimos anos, o que permite desenvolver ou aprimorar técnicas, procedimentos e instrumentos a serem incorporados na prática clínica e de pesquisa.

Embora esses aspectos sejam positivos, este estudo evidenciou que o uso da USG para medir o movimento do osso hioide durante a deglutição ainda não possui procedimentos padronizados. Ainda que o uso da USG com abordagem quantitativa para avaliação de aspectos relacionados à deglutição seja descrito desde a década de 80^(35,36,39)^, a sua transposição para a prática clínica permanece desafiadora, considerando que não é um procedimento usualmente explorado para essa finalidade na formação dos profissionais que atuam com disfagia^(24)^.

Quanto aos parâmetros quantitativos investigados nos estudos incluídos nesta revisão, as medidas de tempo, velocidade e amplitude do movimento, em especial esta última, despontam como os principais desfechos do exame ultrassonográfico. Porém, os equipamentos utilizados e os métodos para obtenção e análise das medidas ainda são bastante heterogêneos ou descritos de modo incipiente, o que compromete a reprodutibilidade e dificulta a transposição do exame para o contexto clínico.

A heterogeneidade dos equipamentos utilizados, incluindo os transdutores, é uma característica que limita comparações entre os estudos, tendo em vista que cada aparelho possui uma taxa de visualização, matriz e frequência diferentes, o que pode impactar na marcação das estruturas de interesse. Quanto ao posicionamento do transdutor, pode-se concluir que as regiões laríngea e submandibular foram as mais utilizadas, sendo a última mais frequente. Ainda que na mesma região, os marcadores anatômicos podem ser diferentes e interferirem no resultado da imagem que será analisada. Chama a atenção o fato de que entre os poucos estudos que estimaram a confiabilidade^(12,14,20,23,27,40)^, os níveis mais altos ficaram entre os que posicionaram o transdutor na região submandibular. Contudo, não é possível comprovar se existe uma relação direta entre o posicionamento do transdutor e a confiabilidade apenas a partir dos dados desta revisão.

A medida de amplitude máxima alcançada pelo osso hioide durante a deglutição foi a mais investigada, ainda que os pontos de referência para obtenção dessa medida tenham sido distintos. Além de marcadores na própria estrutura do osso hioide ou na sombra acústica gerada por ele na imagem ultrassonográfica, foram utilizados a mandíbula ^(20,27,31,40)^, o corpo vertebral cervical^(44)^ e musculaturas adjacentes^(23,29,33,34)^. Algo que pode explicar a amplitude máxima ter sido a medida mais investigada é que ela reflete diretamente o quanto o osso hioide consegue se distanciar do seu ponto de repouso enquanto o indivíduo deglute. Este dado é tradicionalmente considerado relevante no diagnóstico da disfagia orofaríngea, pois está relacionado com a integridade da proteção de vias aéreas inferiores e *clearance* faríngeo^(3-8)^. Além disso, dentre as medidas encontradas, a amplitude é a mais simples de ser analisada em termos operacionais, o que pode ter favorecido seu uso mais frequente.

No que diz respeito às medidas temporais, pode-se observar que a medida mais investigada pelos estudos foi o intervalo de tempo entre o início do movimento do osso hioide para a deglutição até a sua amplitude máxima na trajetória ^(5,14,25-29,35-39,41,42)^. Vale destacar essa medida, pois é um dos indicadores para o risco de penetração ou aspiração^(6)^, visto que, a diminuição no tempo de amplitude pode comprometer o movimento da epiglote prejudicando a proteção da via aérea e, por conseguinte, também dificultar a abertura do segmento faringoesofágico para passagem do bolo alimentar^(44)^.

A maior parte dos estudos incluídos foi realizada com indivíduos saudáveis e investigou as medidas em relação à idade, volume e consistência do bolo alimentar, uma vez que a deglutição passa por mudanças com o avançar da idade^(44)^ e torna-se relevante analisar as inter-relações entre os parâmetros cinemáticos para entender como ocorrem as estratégias de controle motor mediante ajustes de volume e consistência^(29)^.

Os níveis de confiabilidade encontrados, ainda que aceitáveis, não são comparáveis entre os estudos, já que os métodos são distintos. Vale destacar que a análise das imagens ultrassonográficas é perceptivo-visual, requer treinamento e a maioria dos estudos realizou as marcações e análises com processos não automatizados. Todos esses aspectos constituem vieses para os níveis de confiabilidade inter e intra-avaliadores. Nesta revisão não avaliamos os desfechos das medidas, mas salientamos que as mesmas devem ser analisadas e interpretadas considerando as características da população estudada.

## CONCLUSÃO

A medida de movimento do osso hioide durante a deglutição mais investigada por meio da ultrassonografia é a amplitude, seguida por tempo e velocidade. O método de extração das medidas não é padronizado no que se refere aos equipamentos utilizados, posicionamento do transdutor e os procedimentos de análise das imagens.

## Apêndice 1 Estratégias de busca utilizadas nas bases de dados

**Table:**

Base de dados	Descritores	Resultados
Pubmed/Medline	((“deglutition”[MeSH Terms] OR “deglutition”[All Fields] OR “deglutitions”[All Fields] OR “swallowing”[All Fields] OR “swallowings”[All Fields] OR “swallow”[All Fields] OR “swallows”[All Fields] OR “deglutition disorders”[MeSH Terms] OR “deglutition disorders”[All Fields] OR “deglutition disorder”[All Fields] OR “swallowing disorder”[All Fields] OR “swallowing disorders”[All Fields] OR “dysphagia”[All Fields]) AND (“hyoid bone”[MeSH Terms] OR “hyoid”[All Fields]) AND (“ultrasonography”[MeSH Terms] OR “ultrasonography”[All Fields] OR “diagnostic ultrasound”[All Fields] OR “ultrasound”[All Fields] OR “ultrasounds”[All Fields] OR “ultrasound imaging”[All Fields] OR “ultrasonic imaging”[All Fields] OR “medical sonography”[All Fields] OR “ultrasonographic imaging”[All Fields] OR “echography”[All Fields] OR “ultrasonic diagnoses”[All Fields] OR “ultrasonic diagnosis”[All Fields] OR “ultrasonics”[All Fields] OR “sonographic”[All Fields]))	61 resultados
EMBASE	(‘dysphagia’/exp OR ‘swallowing’/exp) AND (‘echography’/exp OR ‘ultrasound’/exp) AND (‘hyoid bone’/exp)	98 resultados
Web of Science	(TS=((“deglutition” OR “deglutitions” OR “swallowing” OR “swallowings” OR “swallow” OR “swallows” OR “deglutition disorders” OR “deglutition disorder” OR “swallowing disorder” OR “swallowing disorders” OR “dysphagia”) AND (“hyoid bone” OR “hyoid”) AND (“ultrasonography” OR “diagnostic ultrasound” OR “ultrasound” OR “ultrasound imaging” OR “ultrasonic imaging” OR “medical sonography” OR “ultrasonographic imaging” OR “echography” OR “ultrasonic diagnoses” OR “ultrasonic diagnosis” OR “ultrasonics” OR “sonographic”)))	51 resultados
Scopus	(TITLE-ABS-KEY (deglutition OR deglutitions OR swallowing OR swallowingsOR swallow OR swallows OR “deglutition AND disorders” OR “deglutition AND disorder” OR “swallowing AND disorder” OR “swallowing AND disorders” OR dysphagia) AND TITLE-ABS-KEY (“hyoid AND bone” OR “hyoid” ) AND TITLE-ABS-KEY (ultrasonography OR “diagnostic AND ultrasound” OR ultrasound OR ultrasounds))	78 resultados

**Table:**

**Legenda:** AVC: acidente vascular cerebral; CCI: coeficiente de correlação intraclasse; OH: osso hioide; USG: ultrassonografia
